# Can an artificial intelligence powered software reliably assess pelvic radiographs?

**DOI:** 10.1007/s00264-023-05722-z

**Published:** 2023-02-17

**Authors:** Gilbert M Schwarz, Sebastian Simon, Jennyfer A Mitterer, Stephanie Huber, Bernhard JH Frank, Alexander Aichmair, Martin Dominkus, Jochen G Hofstaetter

**Affiliations:** 1 Department of Orthopaedics and Trauma-Surgery, Medical University of Vienna, Währinger Gürtel 18-20, 1090 Vienna, Austria; 2 Michael Ogon Laboratory for Orthopaedic Research, Orthopaedic Hospital Vienna Speising, Speisinger Straße 109, 1130 Vienna, Austria; 3 Center for Anatomy and Cell Biology, Medical University Vienna, Währinger Straße 13, 1090 Vienna, Austria; 4 2nd Department, Orthopaedic Hospital Vienna Speising, Speisinger Straße 109, 1130 Vienna, Austria; 5 School of Medicine, Sigmund Freud University Vienna, Freudplatz 3, 1020 Vienna, Austria

**Keywords:** Artificial intelligence, Pelvis, Hip, Radiograph, Hip dysplasia, Femoroacetabular impingement

## Abstract

**Purpose:**

Despite advances of three-dimensional imaging pelvic radiographs remain the cornerstone in the evaluation of the hip joint. However, large inter- and intra-rater variabilities were reported due to subjective landmark setting. Artificial intelligence (AI)–powered software applications could improve the reproducibility of pelvic radiograph evaluation by providing standardized measurements. The aim of this study was to evaluate the reliability and agreement of a newly developed AI algorithm for the evaluation of pelvic radiographs.

**Methods:**

Three-hundred pelvic radiographs from 280 patients with different degrees of acetabular coverage and osteoarthritis (Tönnis Grade 0 to 3) were evaluated. Reliability and agreement between manual measurements and the outputs of the AI software were assessed for the lateral-center-edge (LCE) angle, neck-shaft angle, sharp angle, acetabular index, as well as the femoral head extrusion index.

**Results:**

The AI software provided reliable results in 94.3% (283/300). The ICC values ranged between 0.73 for the Acetabular Index to 0.80 for the LCE Angle. Agreement between readers and AI outputs, given by the standard error of measurement (SEM), was good for hips with normal coverage (LCE-SEM: 3.4°) and no osteoarthritis (LCE-SEM: 3.3°) and worse for hips with undercoverage (LCE-SEM: 5.2°) or severe osteoarthritis (LCE-SEM: 5.1°).

**Conclusion:**

AI-powered applications are a reliable alternative to manual evaluation of pelvic radiographs. While being accurate for patients with normal acetabular coverage and mild signs of osteoarthritis, it needs improvement in the evaluation of patients with hip dysplasia and severe osteoarthritis.

## Introduction

The human pelvis is a complex three-dimensional structure, and anatomical alterations of either the acetabulum and/or the proximal femur can lead to micro-instability or femoroacetabular impingement (FAI) [1]. Hip dysplasia and FAI lead to premature osteoarthritis [2]. Despite advances in magnetic resonance imaging such as biochemical cartilage mapping or traction devices [3], anteroposterior pelvic radiographs remain the cornerstone for the evaluation of the hip joint [4]. Obtaining reliable high-quality radiographic images is essential for an accurate diagnosis, disease classification, and surgical decision-making. Various different radiographic parameters have been proposed to describe the complex relationship between the acetabular coverage and geometry of the proximal femur [1, 5]. Pelvic tilt and rotation have been shown to significantly influence these hip parameters to varying degrees [6, 7]. In addition to technical difficulties of obtaining reliable radiographs, correct landmark setting is dependent upon the experience of the reader and often highly subjective, which is reflected by high inter- and intra-rater variabilities throughout the literature [6, 8–10].

Machine learning, a branch of artificial intelligence (AI), has shown promising results in musculoskeletal radiology for the detection of vertebral body compression, developmental dysplasia of the hip, identification of osteoarthritis, and evaluation of lower limb alignment [11–15]. In prior studies, we showed excellent reliability for the automated lower limb alignment analysis on full leg radiographs with native knees as well as total knee arthroplasties [15, 16]. These AI-powered applications could fill the gap of high inter- and intra-rater variability by providing reproducible measurements. However, no data exists on automated evaluation of pelvic radiographs, and it is further unknown if severe osteoarthritis or the degree of acetabular coverage affects the performance of such software. This is the first study to assess the applicability of an AI algorithm as an aid for the evaluation of the hip joint.

The aim of this study was to assess the reliability and agreement of a newly developed AI software for pelvic radiographs. Our hypothesis was that AI algorithms provide reliable measurements for the lateral-centre-edge (LCE) angle, neck-shaft angle, sharp angle, acetabular index, and the femoral head extrusion index.

## Materials and methods

### AI software

**Fig. 1:**
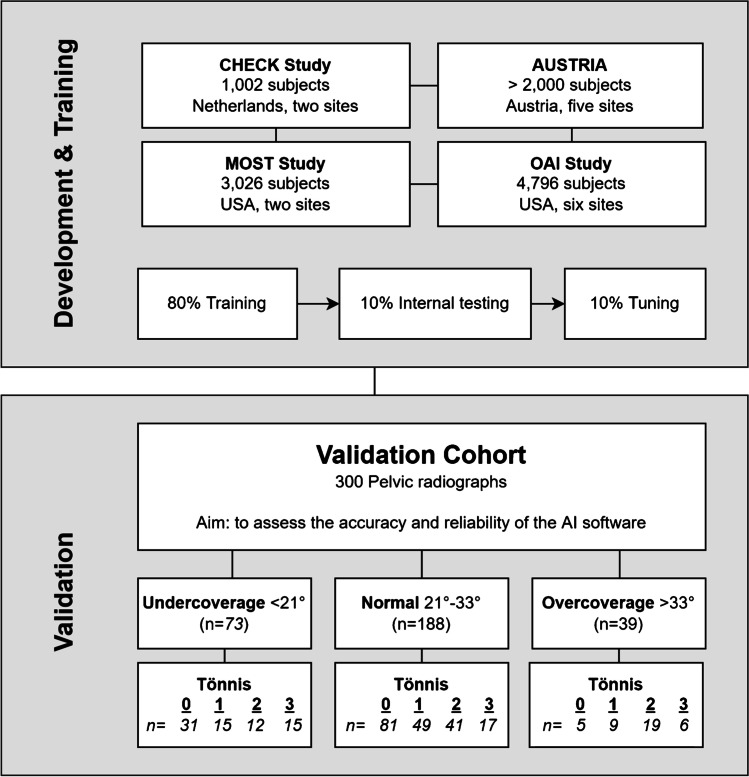
Flowchart of development, training, and validation

**Fig. 2:**
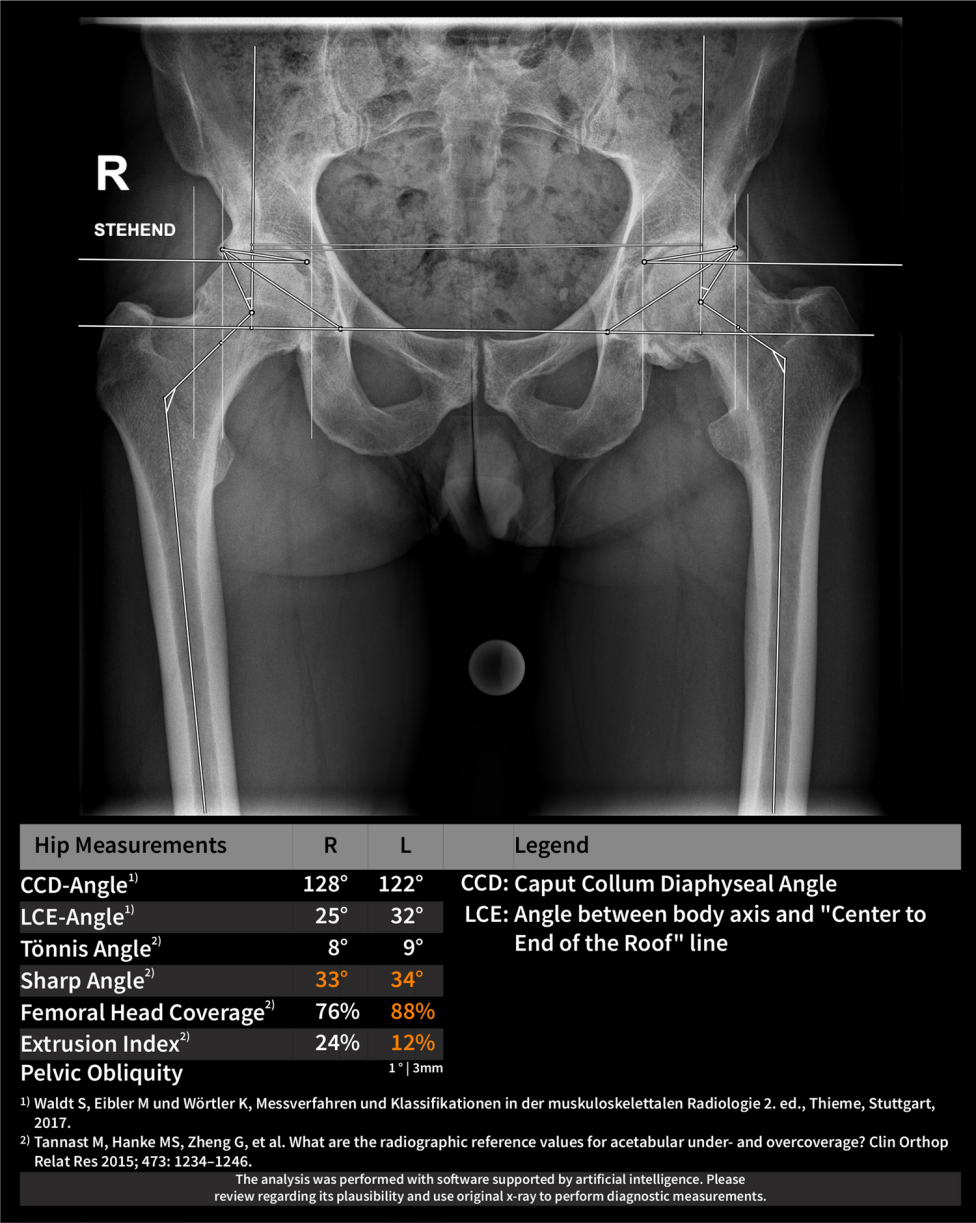
Example output of the AI software for the evaluation of pelvic radiographs

The applied AI software HIPPO (Hip Positioning Assistant 1.03, ImageBiopsy Lab, Vienna, Austria) was built to automate angle measurements on pelvic radiographs. The algorithm was trained on over 10,000 radiographs from the OAI (Osteoarthritis Initiative study; US six-site multi-centre), MOST (Multicenter Osteoarthritis Study, US two-site multi-center), CHECK (Cohort Hip and Cohort Knee study; Netherland single center) studies, as well as five sites in Austria (Fig. 1). A multiple U-Net-based convolutional neural network was engineered, trained, optimized, and validated. The data set was randomly split into 80% training, 10% tuning, and 10% internal test sets. The AI software generates a graphical DICOM output with measured values in tabular form and as an overlay (Fig. 2). In case of failed landmark setting outputs are suppressed. The measurements in this study were performed on a laptop running Ubuntu Linux 18.04 LTS with a 4-core Intel i7 (4600U 2.1 GHz) and 12 GB of RAM, with images stored on an external HDD connected with USB 3.0.

### Correlations between readers and AI software

This study was approved by the local ethics committee (EK: 47/2020). Three hundred pelvic radiographs of 280 patients (191 female, 89 male) with a mean age of 51.9 years (range 16–89) from the institutional image database were included in this study. All images were taken either with the Philips DigitalDiagnost (Philips GmBh, Hamburg, Germany) or Siemens Luminos (Siemens Healthcare GmbH, Erlangen, Germany) fluoroscopy system. All patients were positioned anteroposterior in a standing position with the legs 15° internally rotated and the detector in direct contact to the patient’s body. The central beam was directed to the midpoint of the symphysis, and the film focus distance was 150 cm. For correct length-measurements, a 25-mm calibration ball was added to each radiograph. Cut-off values for pelvic tilt and rotation were applied according to the threshold values of Tannast et al. [6]. Radiographs were repeated if these values were exceeded.

To test the AI algorithm’s ability of detecting structural diseases, a wide range of hips with under- (LCE < 21°), over- (LCE > 33°), and normal (LCE 21–33°) coverage were chosen (Fig. 1). Different degrees of osteoarthritis (Tönnis grades 0 to 3) were also included as suspected AI performance would depend upon the quality of the image. Three orthopaedic surgeons, who routinely perform hip annotations, measured each radiograph using mediCAD® v6.0 (Hectec GmbH, Landshut, Germany). They were blinded to the others and the results from the AI software. The following parameters were measured: LCE angle, neck-shaft angle, sharp angle, acetabular index, and the femoral head extrusion index. We calculated the intraclass-correlation (ICC) between the readers and compared the mean results to the output of the AI software. Based on the minimal detectable change results from Mast et al. [10], the following reference values were chosen: For the LCE angle, sharp angle, and acetabular index, mean absolute differences of 3° between the readers were accepted. For the neck-shaft angle, absolute difference of 5°, and for the femoral head extrusion index, differences of 5% were accepted [10]. In case of wider variances, the correct values were chosen on consensus between all three readers and in consultation with the senior author, who was blinded to the initial measurements of the three readers. Furthermore, two different timings were measured for each radiograph evaluation: (1) the time needed for manual evaluation of each radiograph and (2) the time needed for checking the AI software outputs. (1) The time needed for manual evaluation with mediCAD® was defined as the period between opening the DICOM image, manually setting each landmark and saving it. (2) The time needed for checking the AI software output was defined as the period between opening the DICOM output and record the findings as well as erroneous landmarks.

### Statistics

We employed descriptive statistics, including mean (M), standard deviation (SD), and percentage. We allocated the measured results into reader 1, reader 2, and reader 3, mean of all three readers and their consensus (= ground truth) as well as AI software measurements. Statistical significance was considered for *p* values ≤ 0.05, and Bonferroni correction was applied in multiple testing. The ICC was calculated to assess conformity between the AI software and our manual reads, as well as between the three readers (two-way mixed, single measure model, absolute agreement: ICC3.1). ICC agreement rates were defined as follows: ≥ 0.9 excellent; ≥ 0.75–0.89 good; ≥ 0.5–0.74 moderate; and < 0.49 poor-reliability. The standard error of measurement (SEM) was calculated as $SEM(agrement)=\sqrt{\left({\sigma}_{pt}^2+{\sigma}_{residual}^2\right)}$ as previously reported [10, 17]. We tested the interchangeability index of the AI software compared to the manual reads, where *γ* represents the equivalence index, an estimate of the difference in measurement variability between a reference standard (R), and a new method (T) [18]. The statistical analyses were performed with SPSS 25® (IBM Corp. Released 2018. IBM SPSS Statistics for Windows, Version 25.0. Armonk, NY, USA) and an Excel spreadsheet (Excel 365; Microsoft Inc, Redmond, WA, USA).

## Results

**Fig. 3:**
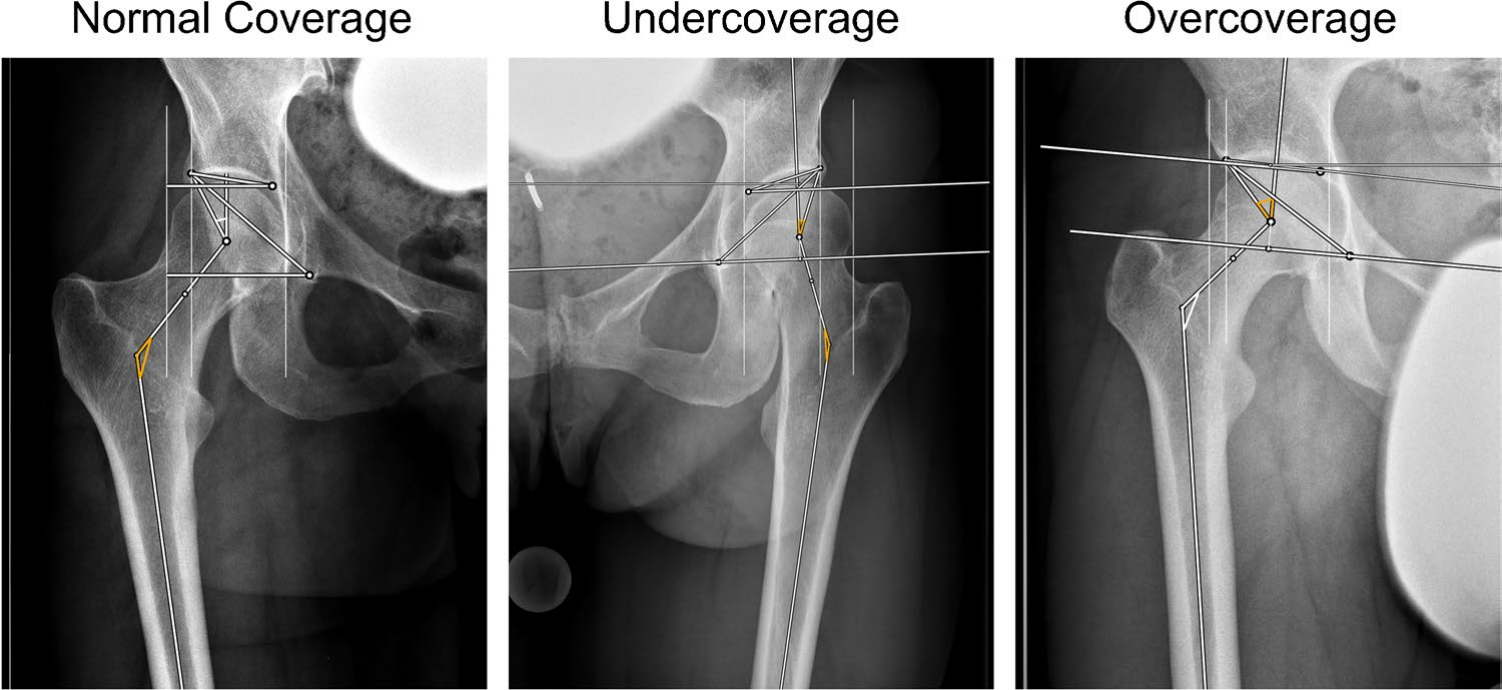
Examples of correct landmark setting for normal acetabular coverage, acetabular undercoverage and acetabular overcoverage

The AI Software provided reliable results in 94.3% (283/300). Examples of reliable outputs are presented in Fig 3. In six cases (2%), no output was provided, and eleven cases (3.6%) had to be excluded due to failed landmark setting based on visual inspection. The neck-shaft angle was affected in eight, the lateral sourcil in three cases, and the center of rotation in one case. Overall, 283 pelvic radiographs were included in the final statistical analysis. Checking the AI output alone (15.8 ± 4.9 s) was ten times faster than manual measurements (171.0 ± 48.5 s, *p* < 0.001).

### Correlations between readers and AI software

**Fig. 4:**
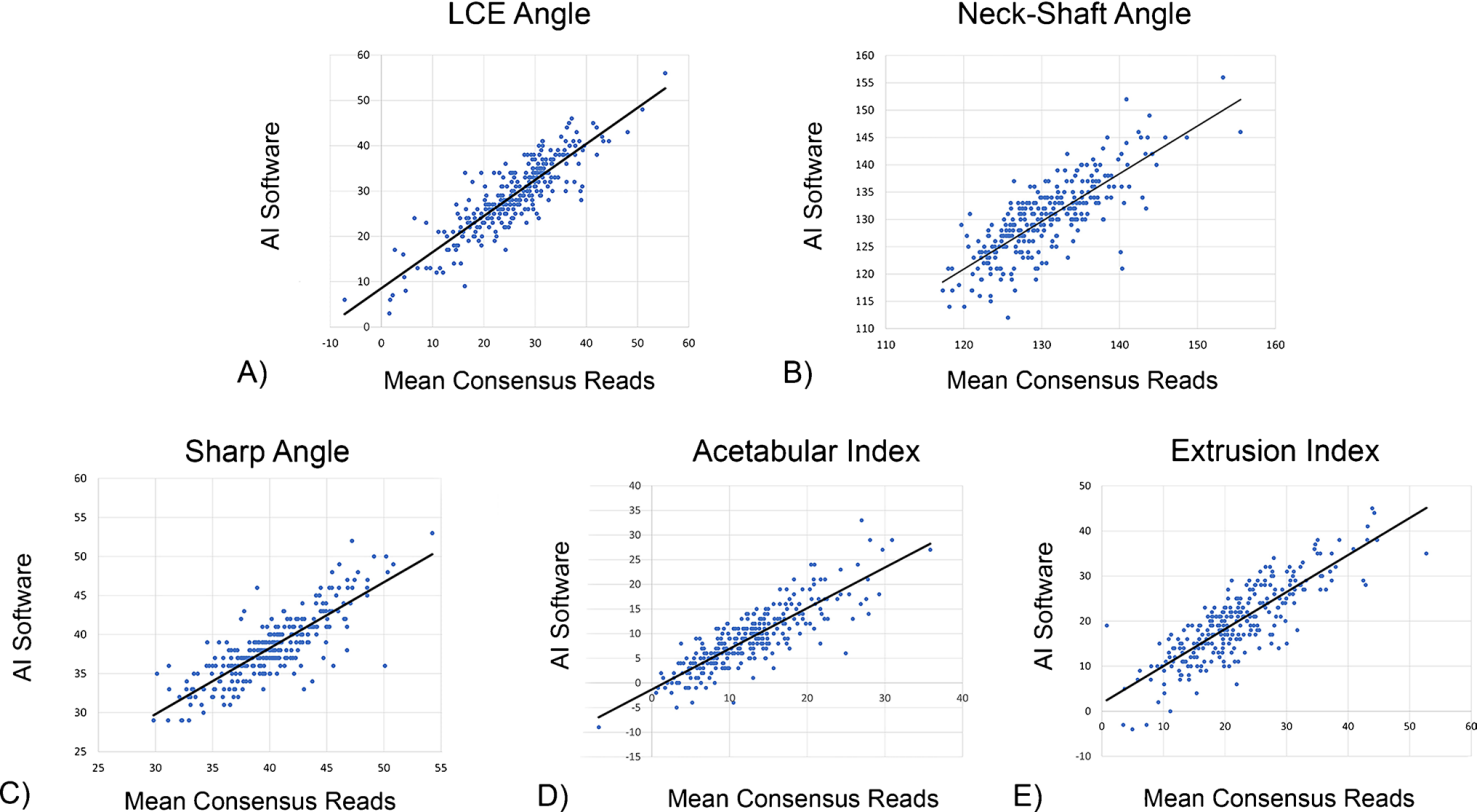
Linear regression graph for the LCE angle (**A**), neck-shaft angle (**B**), sharp angle (**C**), acetabular index (**D**), and femoral head extrusion index (**E**)

Correlation between the AI software and manual measurements revealed moderate to good results for all values (ICC = 0.73–0.80). ICC values for inter-rater reliability were similar (0.69–0.86) to the results between the AI software and the manual reads. The interchangeability (γ) values ranged from 0.3° for the neck-shaft angle to 3.3° for the LCE angle. Detailed results for the mean values, the interchangeability (γ), and the ICC are presented in Table 1. Linear regression graphs can be found in Fig. 4A–E.

**Table 1:** Mean values for the AI software and the consensus reads as well as their interchangeability index (γ) and ICC

	Parameter	Radiographic measurements		Present study			Literature [8-10, 18]	
		AI software	Mean consensus reads	γ (95%-CI)	ICC AI vs. reader	ICC* Inter-rater	ICC Inter-rater	ICC Intra-rater
Total (*n* = 283)	LCE angle	29.1 ± 8.0	25.7 ± 8.7	3.3 (2.7–4.0)	0.80	0.86	0.73–0.92	0.86–0.97
	Neck-shaft angle	130.1 ± 7.0	130.5 ± 6.4	0.3 (– 1.4–2.6)	0.78	0.71	0.58–0.80	0.76–0.95
	Sharp angle	38.4 ± 4.2	40.2 ± 4.2	– 0.7 (-2.4–0.1)	0.75	0.69	0.63–0.82	0.55–0.84
	Acetabular index	8.9 ± 6.2	12.4 ± 6.4	2.6 (2.0–3.5)	0.73	0.76	0.45–0.82	0.73–0.95
	Femoral head extrusion index	19.9 ± 8.2	22.0 ± 8.3	2.9 (2.3–3.7)	0.80	0.86	0.83–0.91	0.73–0.96

*Inter-rater reliability of the original annotations prior to the consensus reads

Overall SEM values ranged from 2.2° for the sharp angle to 3.9° for the LCE angle (Table 2). Hips with acetabular undercoverage had higher SEM values for the LCE angle (5.2° vs. 3.4°), sharp angle (4.2° vs. 2.3°), and extrusion index (4.2% vs. 3.5%). Hips with acetabular overcoverage showed similar SEM values compared to hips with normal coverage. All SEM values became worse with increasing Tönnis grades. The SEM value for the LCE angle changed from 3.3° (Tönnis 0) to 5.1° (Tönnis 3), the neck-shaft angle from 2.2° (Tönnis 0) to 4.7° (Tönnis 3), the acetabular index from 2.6° (Tönnis 0) to 4.3° (Tönnis 1 and 3), and the femoral head extrusion index from 3.3% (Tönnis 0) to 5.3% (Tönnis 3). Only the sharp angle showed consistent results for all degrees of osteoarthritis.

**Table 2:** Standard error of measurement (SEM) overall and for hips with different degrees of acetabular coverage (left) and osteoarthritis (right)

	Overall	Acetabular coverage			Tönnis grade			
		Normal coverage	Under coverage	Over coverage	0	1	2	3
	*n* = 283	*n* = 180	*n* = 66	*n* = 37	*n* = 116	*n* = 67	*n* = 69	*n* = 31
LCE angle (°)	3.9	3.4	5.2	3.5	3.3	3.9	4.3	5.1
Neck-shaft angle (°)	3.1	3.0	3.2	3.7	2.2	3.4	3.3	4.7
Sharp angle (°)	2.2	2.3	4.2	1.9	2.1	2.3	2.2	2.1
Acetabular index (°)	3.5	3.4	3.7	3.9	2.6	4.3	3.7	4.3
Femoral head extrusion index (%)	3.7	3.5	4.2	3.6	3.3	3.1	4.2	5.3

Normal coverage = LCE 21–33°, undercoverage = LCE < 21°, overcoverage = LCE > 33°

**Fig. 5:**
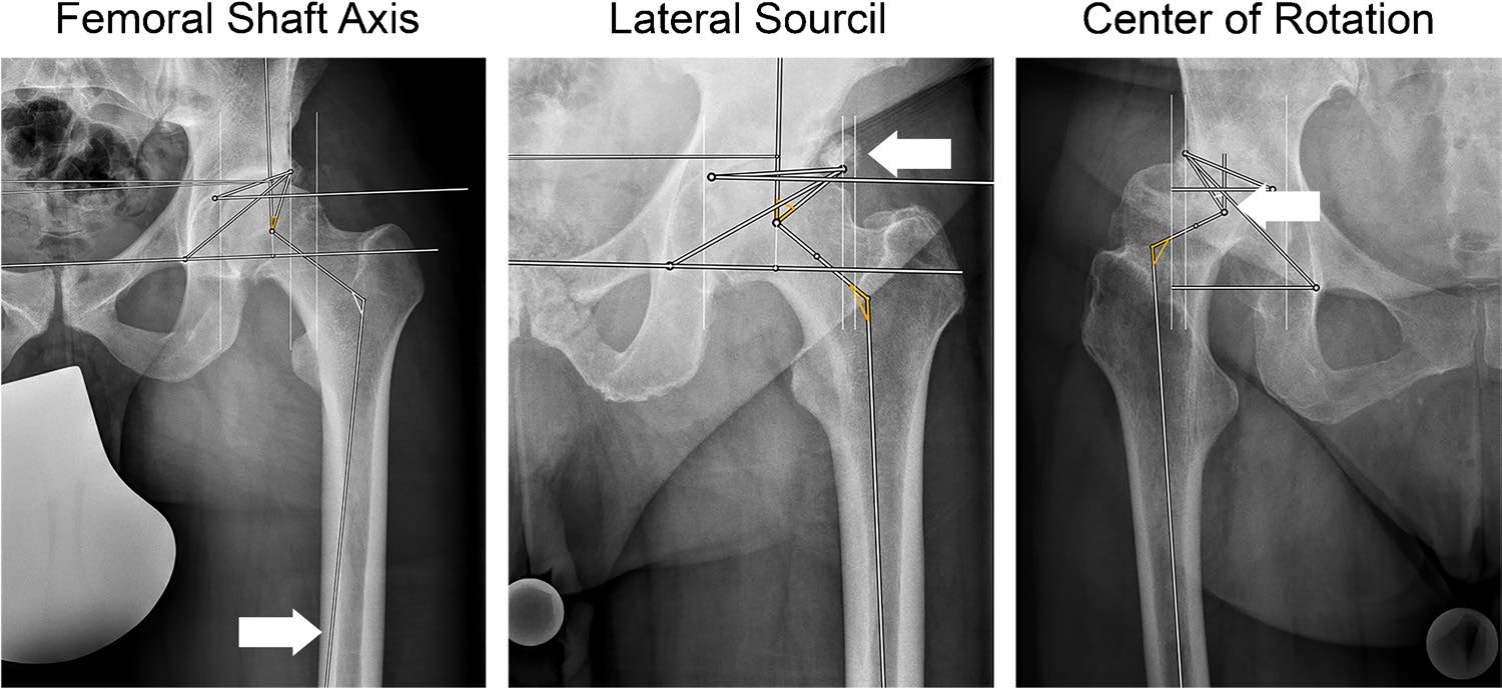
Example radiographs of erroneous landmark setting. Left, failed femoral shaft axis detection; middle, the lateral sourcil was erroneously set at an osteophyte; right, failed detection of the center of rotation and the femoral neck

AI performance for the LCE Angle was best for overcovered and normal covered acetabula with Tönnis grade 0 (SEM = 3.1°). Worst results were seen for undercovered and severe arthritic hips (SEM = 7.4°). Similarly, AI performance for the neck-shaft angle and acetabular index was best for hips with normal coverage and osteoarthritis. The sharp angle showed consistent results for all combinations, and the femoral head extrusion index had particularly bad results for hips with severe osteoarthritis and under- or overcoverage. Contrary to that, hips with normal acetabular coverage had consistent results for the femoral head extrusion index for all degrees of osteoarthritis. Detailed results can be found in Table 3 and examples of erroneous landmark setting in Fig 5.

**Table 3:** Standard error of measurement (SEM) of different combinations of acetabular coverage and degrees of osteoarthritis

		Normal 21–33°		Undercoverage < 21°		Overcoverage > 33°	
	Tönnis	*n*	SEM	*n*	SEM	*n*	SEM
LCE angle (°)	0	80	3.1	31	3.9	5	1.9*
	1	46	3.8	12	4.7	9	3.8
	2	40	3.7	12	6.7	17	3.6
	3	14	3.0	11	7.4	6	5.0
Neck-shaft angle (°)	0	80	2.1	31	2.7	5	2.2
	1	46	3.3	12	3.7	9	4.1
	2	40	3.5	12	2.6	17	3.8
	3	14	5.0	11	4.9	6	5.0
Sharp angle (°)	0	80	2.2	31	2.1	5	2.2
	1	46	2.6	12	1.5	9	2.1
	2	40	2.6	12	1.9	17	1.4
	3	14	1.5	11	2.4	6	3.2
Acetabular index (°)	0	80	2.4	31	2.8	5	3.8
	1	46	4.4	12	4.6	9	3.7
	2	40	3.6	12	4.1	17	3.7
	3	14	3.9	11	4.7	6	5.4
Femoral head extrusion index (%)	0	80	3.4	31	2.7	5	4.5
	1	46	3.0	12	3.2	9	3.7
	2	40	4.3	12	5.9	17	2.1
	3	14	3.8	11	6.5	6	6.2

Best results for each parameter are marked in bright grey and worst values in dark grey. *SEM value might be too low due to the small number of radiographs in this subgroup

## Discussion

This is the first study to evaluate the applicability of a newly developed AI algorithm for the assessment of pelvic radiographs. We showed that automated analysis using an AI-powered software is a reliable alternative to manual measurements and provided reliable results in 94.3% of all cases. In terms of reliability, presented ICCs were comparable to the results of the literature. All ICC results were within the published literature or slightly better. Only the ICC for the femoral head extrusion index was worse than previously reported. The ICC of the LCE angle (ICC = 0.80) was in-between the values of Mast et al. [10] (ICC = 0.73) and Tannast et al. [9] (ICC = 0.92). The ICC of the neck-shaft angle (ICC = 0.78) was better than the values presented by Nelitz et al. [8] (ICC = 0.72) and Mast et al. [10] (ICC = 0.58). The sharp angle and acetabular index were also both within the presented values of the literature. Only the femoral head extrusion index showed slightly worse results compared to the literature (ICC = 0.80 vs. 0.83–0.91) [8, 9].

Agreement rates are defined as the degree of which repeated measurements vary for individuals [19]. Hips with normal acetabular coverage showed good agreement values for all investigated parameters. For example, the SEM value for the LCE Angle was 3.4° and for the neck-shaft angle 3.1°. These values were comparable to results from the literature [10]. Only the neck-shaft angle in hips with a Tönnis grade 3 showed worse SEM values. However, in the study from Mast et al. [10], only hips from relatively healthy patients with Tönnis grades 0 to 1 were investigated. Cut-off values for reliable software results for each parameter can be found in Tables 2 and 3. We observed that hips with acetabular overcoverage (LCE > 33°) generally had slightly worse agreement values compared to hips with normal coverage (LCE 21° to 33°). With increasing degrees of osteoarthritis, SEM values for these hips became even larger with the worst SEM values seen for Tönnis grade 3 with 6.2% for the femoral head extrusion index or 5.0° for the LCE angle. Agreement for hips with acetabular undercoverage was good for the neck-shaft angle and the sharp angle. However, the SEM value for the LCE angle ranged between 3.9° for hips with Tönnis Grade 0 and 7.4° for severe osteoarthritic hips (Tönnis 3), which lessens the applicability of the AI software in patients with hip dysplasia and severe osteoarthritis.

A common issue in AI algorithms is the black box phenomenon, which refers to a system in which only the input and outputs are visible, but not the internal mechanics [7]. Although, as a consequence, no adequate failure modes were presented, we could identify two major issues when visually comparing the manual reads with the AI outputs. The AI algorithm set the lateral acetabular sourcil in general more lateral and the centre of rotation often too medial. Although differences were small, these differences obviously had a significant impact on parameters like the LCE angle or acetabular index. This is further supported by the high inter- and intra-rater variabilities of these values in the literature [10].

From our perspective, there are a few limitations to the applicability of such software. First, the overestimation of the LCE angle might lead to undiagnosed cases of hip dysplasia. However, as previously described, definitive diagnosis should be based on careful synthesis of physical examination and detailed history and not solely lean on one parameter [5, 10]. Second, while correcting for pelvic obliquity, the AI software does not take pelvic tilt and rotation into account. Previously published programs like Hip^2^Norm aimed to correct for that by taking the individual apparent rotation and tilt into account [20]. As shown by Tannast et al. [7], almost all angles are affected by the pelvic position, and severely rotated radiographs might show wrong values. These two major limitations must be addressed in future software updates to show reliable results for all degrees of acetabular coverage.

The primary limitation to the generalization of our results is our chosen study population. Although we included significantly more radiographs than in previous studies on inter- and intra-rater reliability, our subgroups became relatively small [8, 10, 21]. Furthermore, all included images were sourced from a single site and two radiography devices with fixed distances between film and focus. We excluded severe deformities of the femoral head, because suspected intra-rater variability would be too high in these cases. All three readers had the same level of experience for annotating pelvic radiographs. The bias was mitigated by consulting the senior author for our consensus reads in case of contradicting measurements. We believe that there was no bias in our study, as presented inter-rater values were similar to the published literature [9, 10]. Other limitations concerned the AI software itself, which only presented the described parameters and corrected for pelvic obliquity but not for tilt and rotation. Furthermore, the AI software requires no input from the clinician and therefore must always be reviewed for safety and accuracy.

AI has enormous potential in the field of orthopedics [22]. The ability to evaluate large datasets in a standardized way offers entirely new possibilities by increasing the power of previously undersized studies. However, assessment of pelvic radiographs, as presented here, is only the first step in the broad applicability of machine learning. Future AI algorithms might help developing new parameters and improve the understanding of the natural course of hip dysplasia, FAI, and osteoarthritis of the hip.

## Conclusion

Presented AI algorithm is a reproducible alternative to manual evaluation of pelvic radiographs. While performance needs to be improved for hips with acetabular undercoverage and severe osteoarthritis, it provides reliable outputs for patients with normal acetabular coverage and/or only mild signs of osteoarthritis.

## Data Availability

The authors confirm that the data supporting the findings of this study are available within the article. Additional data is available from the corresponding author (Gilbert Manuel Schwarz) on request.

## Declarations

### Disclaimer

The funders of this study had no role in study design; in the collection, analysis, and interpretation of data; in the writing of the report; and in the decision to submit the paper for publication.
